# Discovery in clinical and translational medicine

**DOI:** 10.1002/ctm2.568

**Published:** 2021-10-18

**Authors:** Diane C Wang, Xiangdong Wang

**Affiliations:** ^1^ Department of Emergency Medicine Sunshine Coast University Hospital Sunshine Coast Australia; ^2^ Department of Pulmonary and Critical Care Medicine Fudan University Zhongshan Hospital Shanghai P. R. China

## Abstract

With the rapid development of biotechnologies and deep improvement of knowledge, “Discovery” is the initial period and source of innovation of clinical and translational medicine. The international journal of *Clinical and Translational Discovery* serves to highlight unknown or unclear aspects of clinical and translational medicine‐associated knowledge, technologies, mechanisms, and therapies (https://onlinelibrary.wiley.com/journal/27680622). The Discovery aims to define the interaction between genes, proteins, and cells, and explore molecular mechanisms of intercommunication and inter‐regulation. More discoveries of technologies and equipment are expected to improve method sensitivity, specificity, stability, analysis, and clinical significance. The first priority of *Clinical and Translational Discovery* is to turn gene‐, protein‐, drug‐, cell‐, and interaction‐based discoveries into health advancements. *Clinical and Translational Discovery* highly focuses on the discoveries of biological therapies and precision medicine‐based therapy elicited from computational chemistry, DNA libraries, target‐dependent small molecular drugs, high‐throughput screening, vaccination, immune therapy, cell implantations, gene editing, and RNA‐ or protein‐based inhibitors. Thus, *Clinical and Translational Discovery* sincerely welcome you to join and share the rapid development and future successes to come.

Translational and clinical science constitutes a critical part of biomedicine and is one of the vital novel interdisciplinary approaches to “translate the remarkable scientific innovations into health gains,” a milestone concept headlined by Zerhouni.^1^ The translation from discovery to health care benefits and promotion of human wellbeing is an important process to improve the quality of human life. Such translation is expected to provide more precise molecular knowledge and understanding prior to disease development, progression, response to therapy, and prognosis, identify clinical phenome‐specific, disease‐specific, and therapy‐specific biomarkers and targets, and define more opportunities and challenges within biomedical research. Out of basic, translational, clinical, and public health sciences, the “transformation of translational clinical science” was suggested to delineate new landscapes of disease and health, characterize decisive shifts from acute to chronic diseases and supply immediate controls from epidemic outbreaks and infections to new balances of ecology.^2^ Of those concepts, clinical and translational medicine was defined as the “clinical potential and application of translational research and science to improve the understanding of mechanisms and therapies of human diseases,” and mainly focuses on the development of disease‐specific biomarkers and therapeutic strategies to monitor and cure disease.^3^ Furthermore, *Clinical and Translational Discovery* serves to highlight unknown or unclear aspects of clinical and translational medicine‐associated knowledge, technologies, mechanisms, and therapies. *Clinical and Translational Discovery* (https://onlinelibrary.wiley.com/journal/27680622), with a close relationship and collaboration with the journal of *Clinical and Translational Medicine* (https://onlinelibrary.wiley.com/journal/20011326), is a new pathway to clinical practice and health care.

The “Discovery” plays an important and decisive role in the development of translational science and medicine, clinical and molecular medicine, and spatiotemporal molecular medicine. “Discovery”, the initial period and source of innovation of clinical and translational medicine, is founded on gene‐based structure and function, regulation and editing, detection and diagnosis, analysis and intelligence, imaging and therapy, and ethics and regulations. For example, disease‐influencing risk factors can be uncovered by monitoring the copy number polymorphism in the gene cluster of chromosome assembly, the neocentromere by detecting variable number tandem repeats in chromosome, and clues of evolutionary reconstruction can be identified by defining the overall organization and methylation patterns of the centromere in a diploid human genome.^4^ The multidimensional genomes provide new approaches to uncover new regulators and mechanisms in nuclear organization, gene transcription regulation, spatiotemporal development, and genome assembly.^5^ Reshaped genome construction causes gene dysfunction through long‐range chromatin contacts between risk loci and putative target genes, leading to molecular phenomes of pathology and revolutionary values in clinical and translational medicine.^6^ Single‐cell nuclear elements contribute to the interaction among transcriptional factors, DNA elements, and genome organization, responsible for regulatory mechanisms of the developmental evolution and spatiotemporal changes of cell molecular phenomics, clonal genotypes, and structural metamorphoses in diseases.^7^

More discoveries of technologies and equipment are expected to improve method sensitivity, specificity, stability, analysis, and clinical significance. With the rapid development of technologies and understanding, the definition and understanding of clinical gene testing have been continuously renewed and extended to include structures and functions of chromosomes and chromatins, DNA and associated regulators, RNA and RNA‐associated factors, and genome dimensions and regulations among various durations, phases, and conditions.^8^ The *Clinical and Translational Discovery* turns discoveries of chromosome/chromatin, DNA, and RNA sequence, structure, and function into clinical application for predicting, diagnosing, monitoring, and prognosing human disease‐specific phenomes, while characterizing severities, durations, stages, and responses to therapy.

One of the aims of *Clinical and Translational Discovery* is to turn protein discoveries into health advancements through highlighting and exploration of protein cryo‐electron microscopy structure, antibody library, enzyme engineering, computational biology, phase separation, and drug development. Of those discoveries, protein‐based biomarkers, targets, and therapies are identified and developed to directly improve the outcomes of patients with chronic, rare, and difficult diseases. For example, more human disease proteome alterations have been mapped and uncovered by combining in‐depth histopathology with proteome characterization, protein levels with multiple localizations, chromosomal and subcellular distributions with organ/tissue function, and protein profiles with clinical phenomes.^9^, ^10^ The package of protein discovery includes the identification of peptide spectrum matches, protein‐coding genes, peptides, sites of phosphorylation and acetylation, crossing networks among multiomics, spatiotemporal localization, mutations, splice isoforms, and variants in diseases. The combination of RNA and protein discoveries has made significant contributions to our understanding of disease and developing new vaccinations and neutralizations in the coronavirus disease 2019 (COVID‐19) pandemic caused by severe acute respiratory syndrome coronavirus 2 (SARS‐CoV‐2). The dynamics of antibody reactivity to the receptor‐binding domain (RBD) of SARS‐CoV‐2, neutralizing activity, and the number of RBD‐specific memory B cells are associated with the disease severity of patients with or without vaccination after infection.^11^ The stabilization and mechanisms of the systemic immune capacity of those particular patients were defined and influenced by the vaccination. This provides solid evidence of therapeutic windows and potentials for clinicians to make clinical decisions of therapy types, precise duration, and solutions of assistance with the prevention, to improve the incidence and prognosis of patients.

Structures performing protein functions can be systemically and precisely synthesized and characterized using progressive technologies of bioengineering. Proteins, as intra‐ and intercellular critical elements, structurally support the frameworks of organelles, cells, and microenvironments, functionally control intercommunication between organelles, cells, and organs, and represent systemic or local tissue(s) pathophysiology. For example, the neuronal cell adhesion molecules in cortical astrocytes, enriched at astrocyte–neuron junctions with a barrier function against cell infiltration, were profiled using an in vivo chemicogenetic approach with a cell‐surface fragment complementation strategy, to explore the function of astrocyte–synapse adhesions, astrocyte contacts‐controlled synapse formation and function, and interactions among proteins.^12^ More discoveries on the transit between cell types/subtypes, between acute and chronic conditions, between chronic disease and cancer, and between tumorigenesis and chromatin/epigenetic instability can be carried out by integrating molecular networks from multiomics and transomics with functions and regulations. Studies on the cryoelectron microscopy structure that functions similar to the major facilitator superfamily domain‐containing protein 2a (MFSD2A) demonstrated that this sodium‐dependent lysophosphatidylcholine symporter has a conserved sodium‐binding site as a potential lipid entry pathway,^13^ which might be a druggable target for new drug identification and development.

The discoveries of biological therapies and precision medicine‐based therapy elicited from computational chemistry, DNA libraries, target‐dependent small molecular drugs, high‐throughput screening, vaccination, immune therapy, cell implantations, gene editing, and RNA‐ or protein‐based inhibitors form the focus of *Clinical and Translational Discovery*. Precision medicine is a concept of a new therapeutic strategy and an approach to discover and develop new drugs and vaccines by integrating clinical and molecular information with the aim to define exact patient populations.^14^ Small‐molecule drugs can be screened and selected by defining the cellular quality control machinery to selectively degrade target proteins and by measuring the induced protein degradation using proteolysis‐targeting chimaeras. Protein‐protein interactions are important processes to recognize different cell functional phenomes and provide new prospects to identify therapeutic targets, screen functional sensitivity and resistance to drugs, and develop new strategies for interventions. The heterogeneity, dynamics, and complexity of protein‐protein interactions offer more opportunities for drug discovery. The understanding of structures, mechanisms, clinical applications, and off‐target activities of genome editing systems through integration of gene sequencing, clinical transomics, and single‐cell biomedicine was proposed as a critical part of clinical precision medicine strategies and multidisciplinary therapy strategies.^15^, ^16^, ^17^, ^18^, ^19^ The specificity and efficiency of on‐target/off‐target sites need to be furthermore clarified during the process of translating gene editing into clinical practice. The clinical application of gene‐edited immune cells or stem cells is a new type of precision medicine‐based or target‐oriented therapy.^20^ The artificial nucleic acid molecules were designed and constructed by combining aptamers binding to two key players of innate immune responses, to inhibit the immune responsiveness, improve the gene‐editing efficiency, and promote inhibitory effects on the growth of cancer cells.^21^

In addition to gene‐gene and protein‐protein interactions, *Clinical and Translational Discovery* will also focus on molecular configurations and regulations of spatiotemporal cell–cell communication and interactions. Cell–cell communication is an important and potential‐laden opportunity to understand and alter biological functions and microenvironmental hemostasis of cells, organs, and intact systems to improve health. Single‐cell sequencing was suggested as an approach to explore molecular regulations during cell–cell communication and discover function‐specific target molecules within intercellular networks.^22^ Single‐cell transcriptomes demonstrate new clusters/subtypes of cells, cell–cell communication, organ parenchymal and nonparenchymal cell lineages, and ecosystem landscapes of microenvironments. Extracellular vesicles are major carriers of extracellular RNA, such as microRNA, lnRNA, and ncRNA, and are responsible for the delivery of intercellular messages and the balance of microenvironmental hemostasis. The comprehensive networks and interactomes of protein–protein or RNA–RNA interactions control the intensity of intercellular signals and regulate the cell–cell interacted axis in organ immunity. Combining bulk and single‐cell RNA sequencing and predicting ligand–receptor pairs of prostate glandular epithelia reveal that luminal cells downregulate the multipotency and activation of basal cells and multipotent basal stem cells by producing TNF and inactivating the Notch, Wnt, and EGFR pathways, indicating that the heterotypic intercellular communication maintains lineage fidelity of stem cells.^23^ Cell–cell communication also plays important roles in understanding spatiotemporal molecular images and medicine.^24^, ^25^

We, as editors of *Clinical and Translational Discovery* and *Clinical and Translational Medicine*, sincerely welcome you to join and share the rapid development and future successes of the journal. We deeply appreciate the scientists who have been involved in the preparation, our colleagues for their efforts and contributions, as well as our Editorial Board members for their endless support. We strongly believe that *Clinical and Translational Discovery* will be a new pathway to gather scientists globally to achieve and transform new discoveries in clinical and translational medicine into worldwide health gains.

## CONFLICT OF INTEREST

The authors declare that there is no conflict of interest.
